# Supplementary material for: Novel digital signatures of tissue phenotypes for predicting distant metastasis in colorectal cancer
Source: Sci Rep. 2018 Sep 12;8:13692. doi: 10.1038/s41598-018-31799-3 (PMC6135776; doi:10.1038/s41598-018-31799-3)
Supplement: Supplementary file 1 — Supplementary materials [file 41598_2018_31799_MOESM1_ESM.pdf]

Novel Digital Signatures of Tissue Phenotypes for Predicting Distant Metastasis in Colorectal Cancer : Supplementary Information

Korsuk Sirinukunwattana, David Snead, David Epstein, Zia Aftab, Imaad Mujeeb, Yee Wah Tsang, Ian Cree, and Nasir Rajpoot

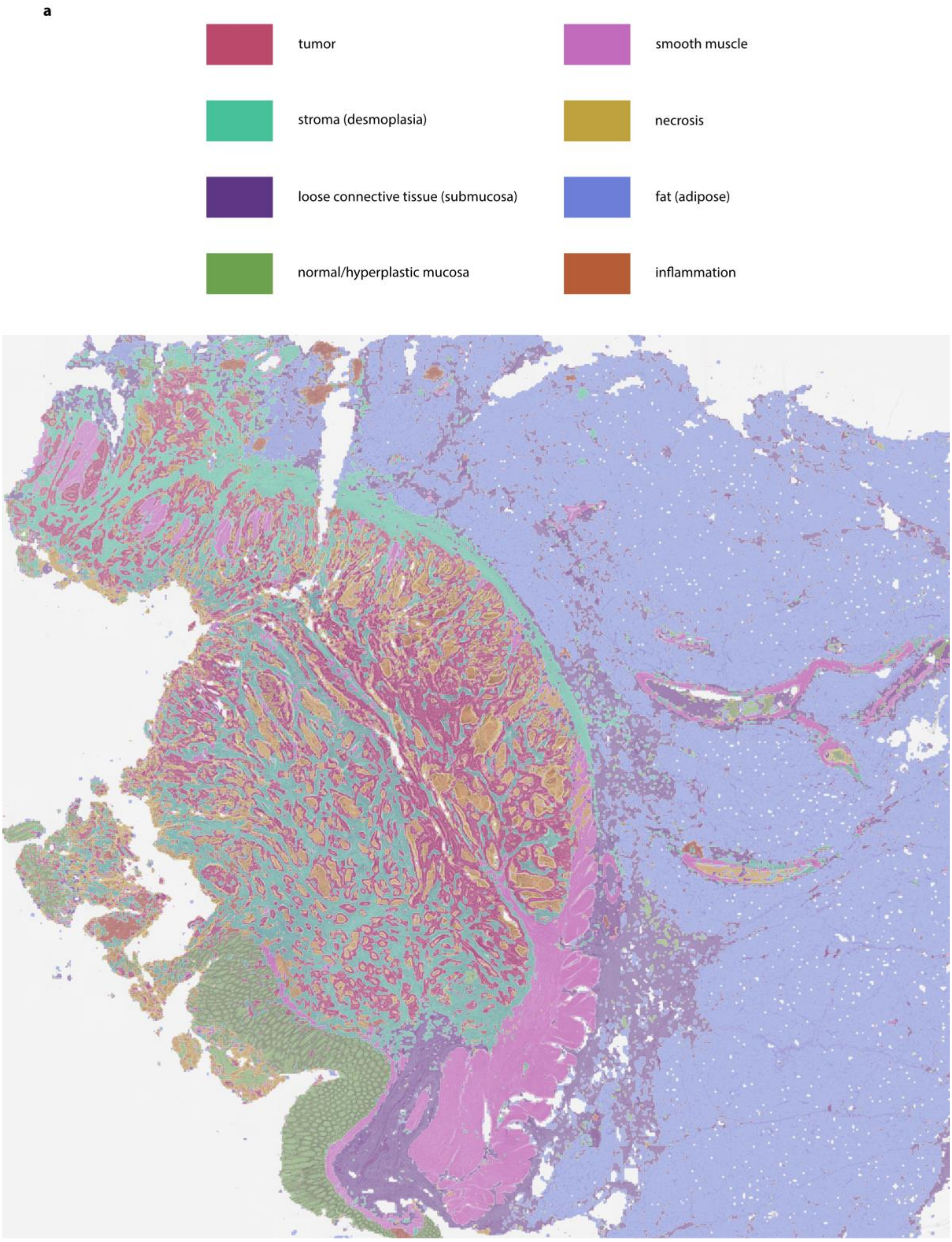

b

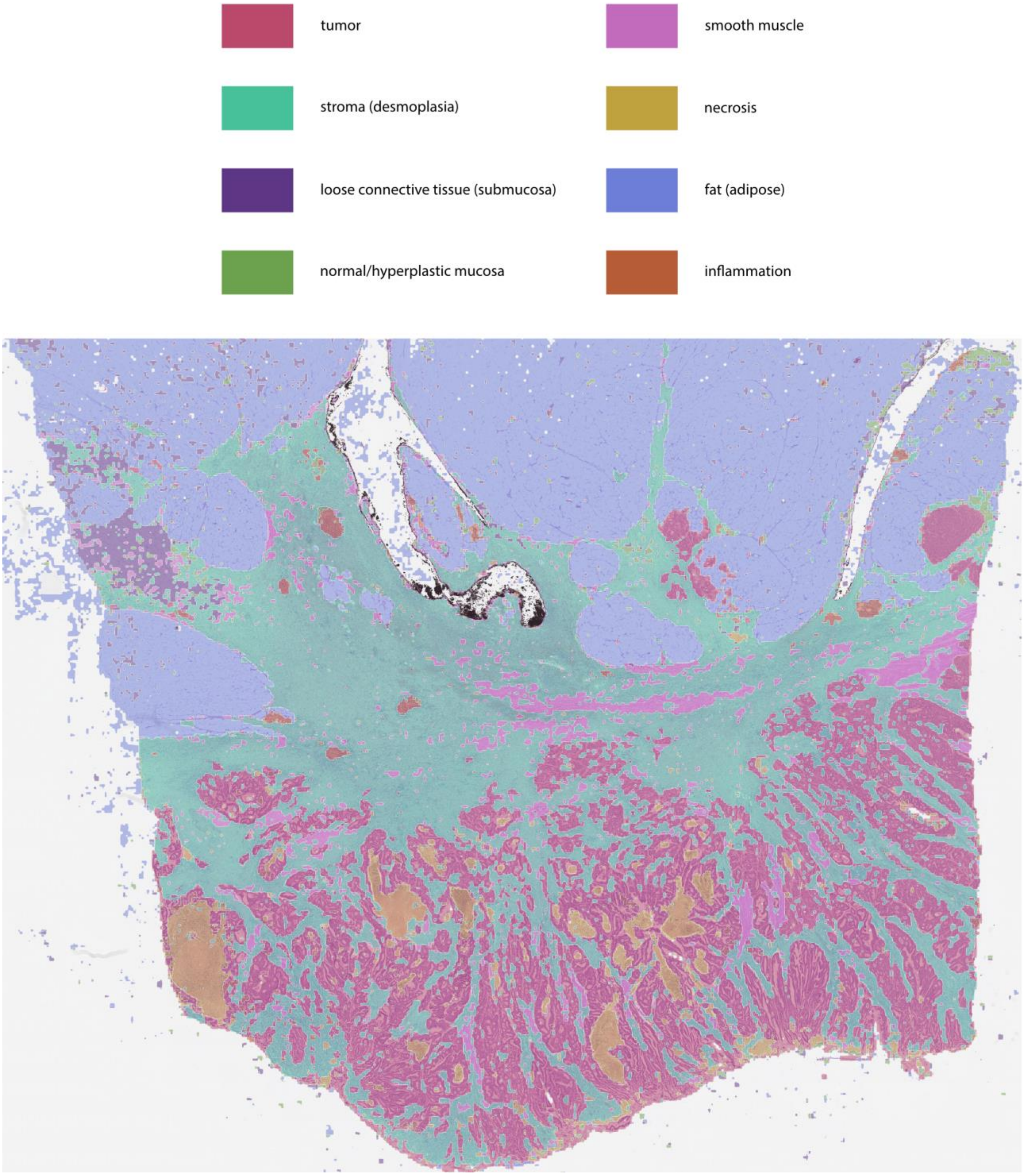
tumor
smooth muscle
stroma (desmoplasia)
necrosis
loose connective tissue (submucosa)
fat (adipose)
normal/hyperplastic mucosa
inflammation

c

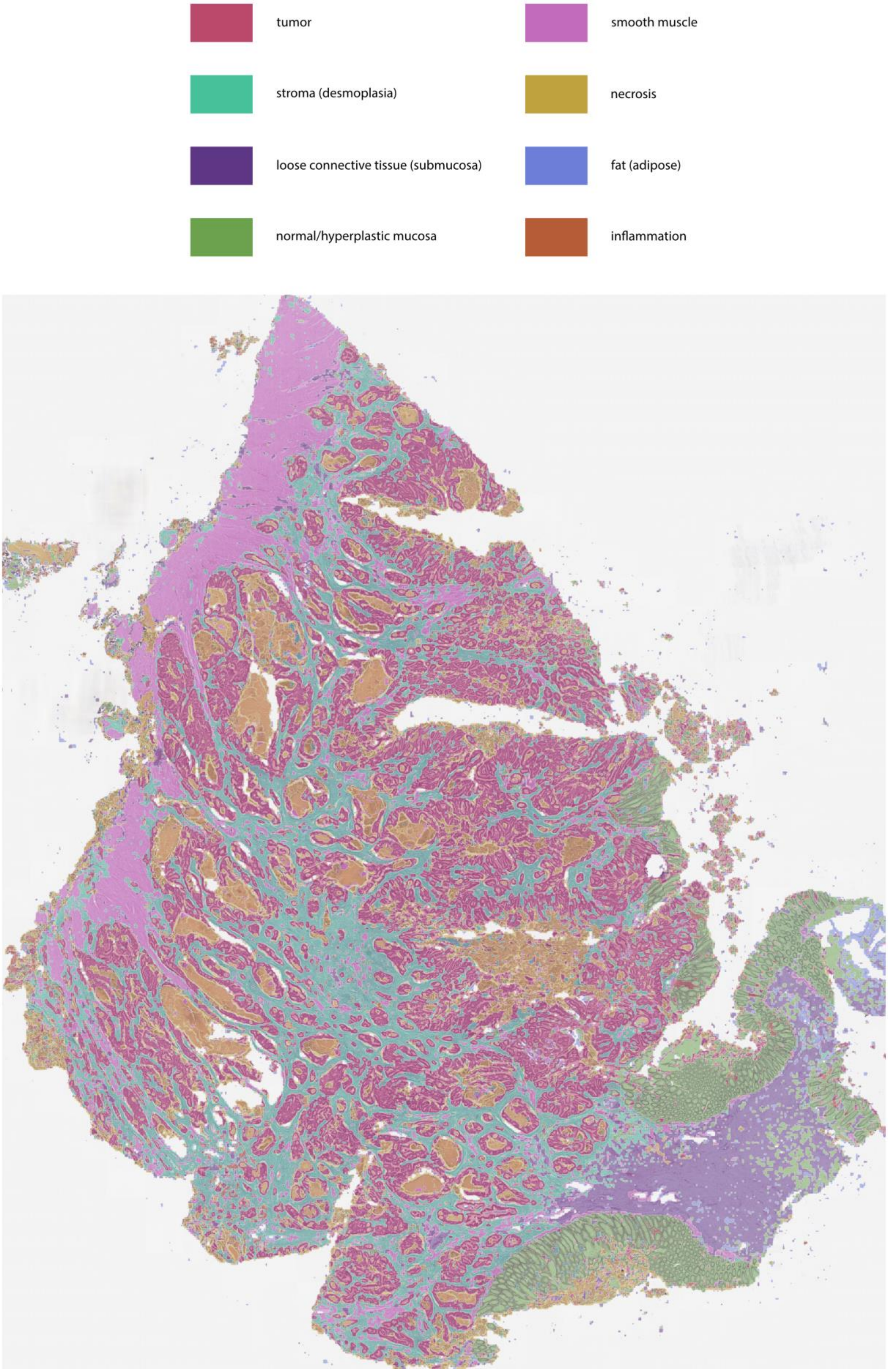
tumor
smooth muscle
stroma (desmoplasia)
necrosis
loose connective tissue (submucosa)
fat (adipose)
normal/hyperplastic mucosa
inflammation

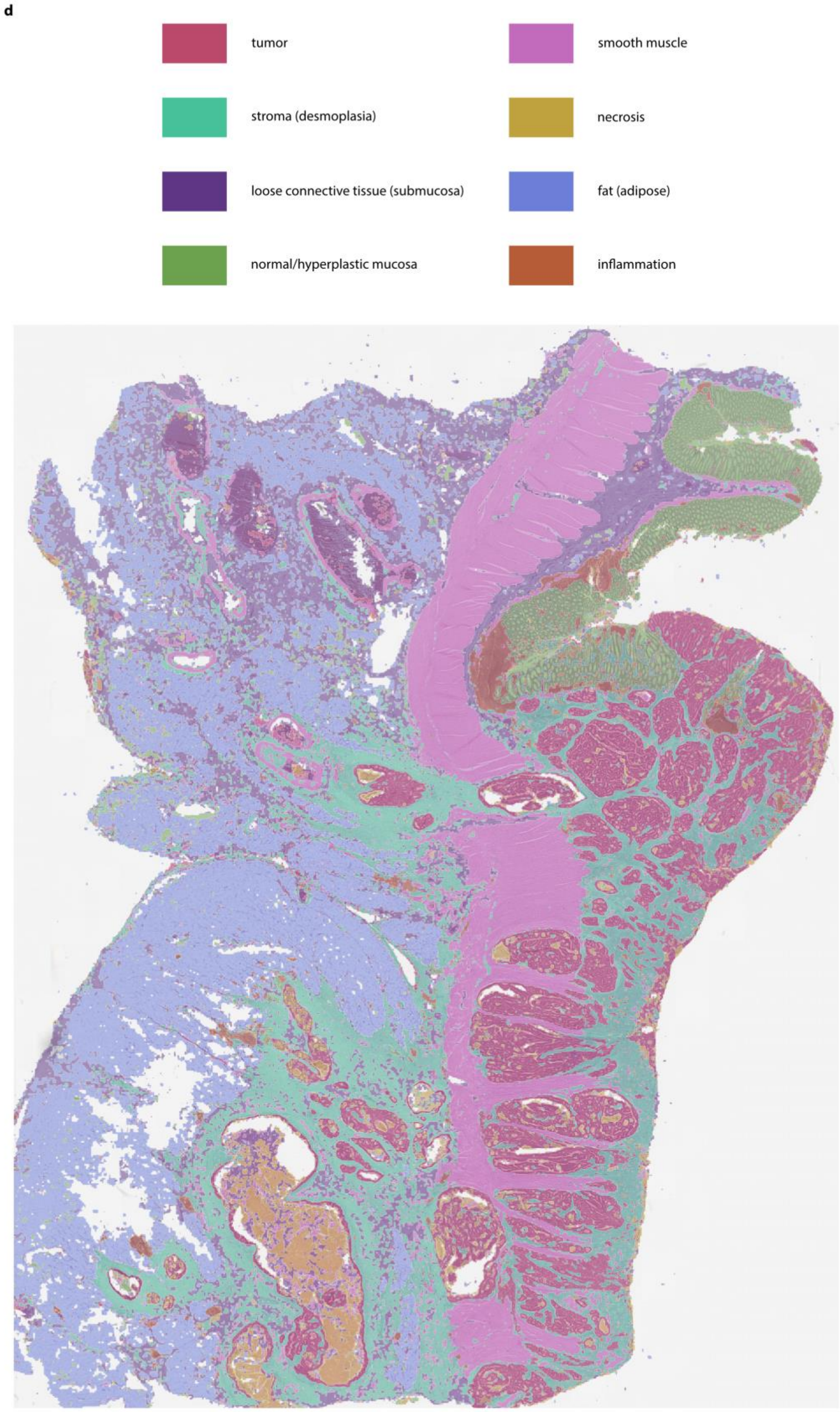

Fig. S1. Example of segmentation results.

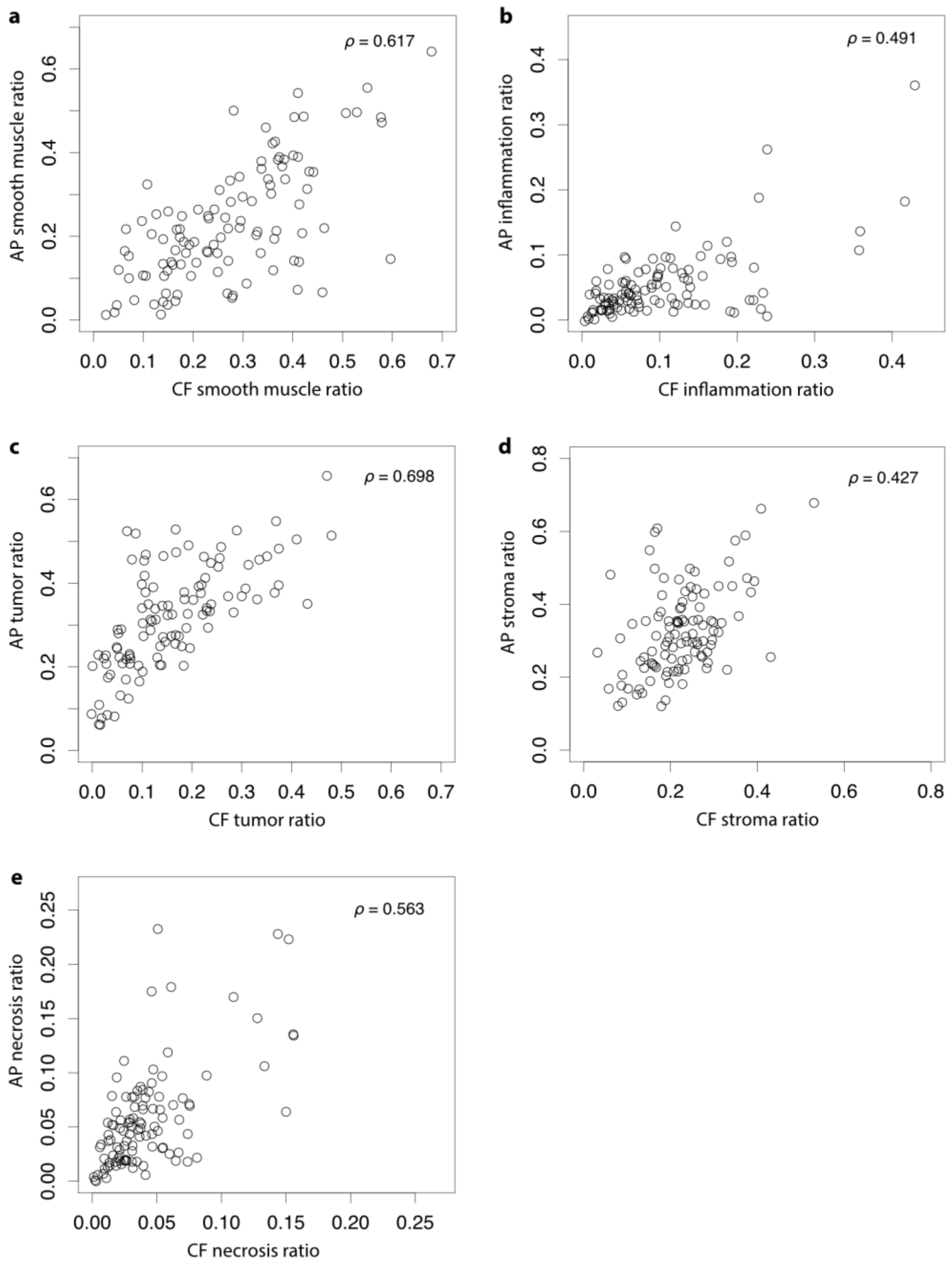

Fig. S2. Correlation between the corresponding CF and AP tissue phenotypic features. The correlation is measured by Spearman correlation coefficient ρ.

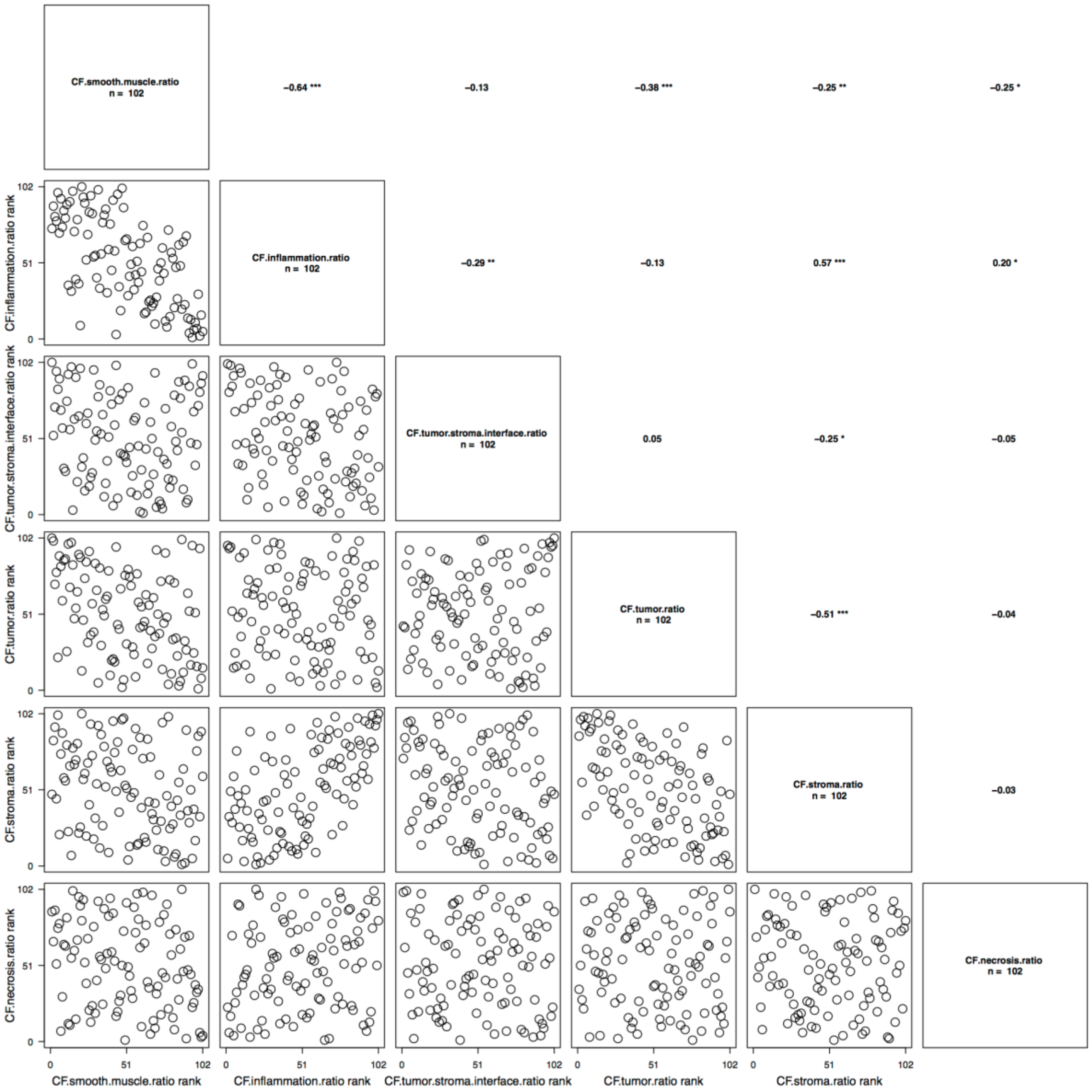

Fig. S3. Correlation plots between the CF tissue phenotypic features. The correlation is measured by Spearman correlation coefficient ρ. Variable ranks are plotted.

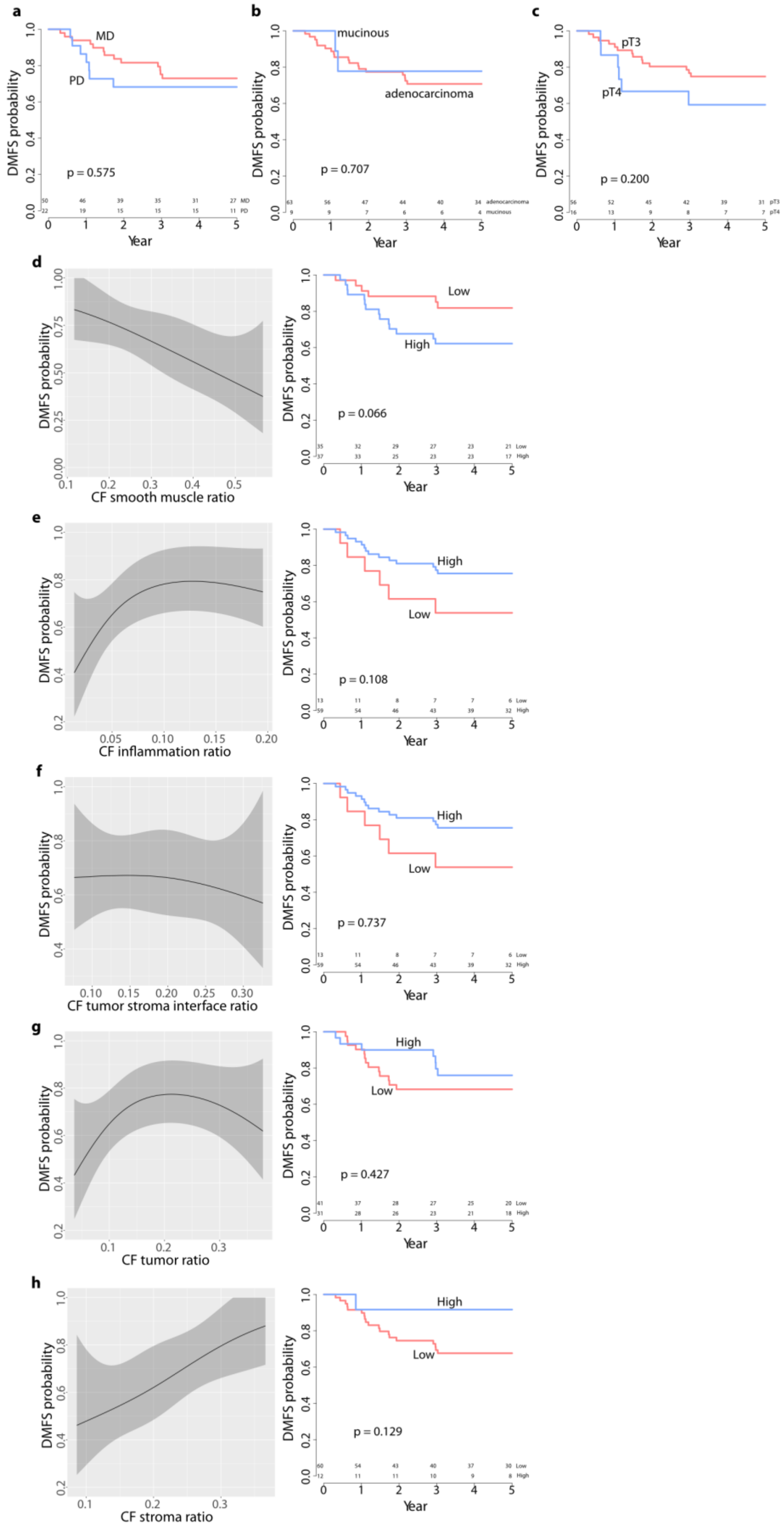
a
MD
PD
p = 0.575
DMFS probability
Year
b
mucinous
adenocarcinoma
p = 0.707
DMFS probability
Year
c
pT3
pT4
p = 0.200
DMFS probability
Year
d
DMFS probability
CF smooth muscle ratio
Low
High
p = 0.066
Year
e
DMFS probability
CF inflammation ratio
High
Low
p = 0.108
Year
f
DMFS probability
CF tumor stroma interface ratio
High
Low
p = 0.737
Year
g
DMFS probability
CF tumor ratio
High
Low
p = 0.427
Year
h
DMFS probability
CF stroma ratio
High
Low
p = 0.129
Year

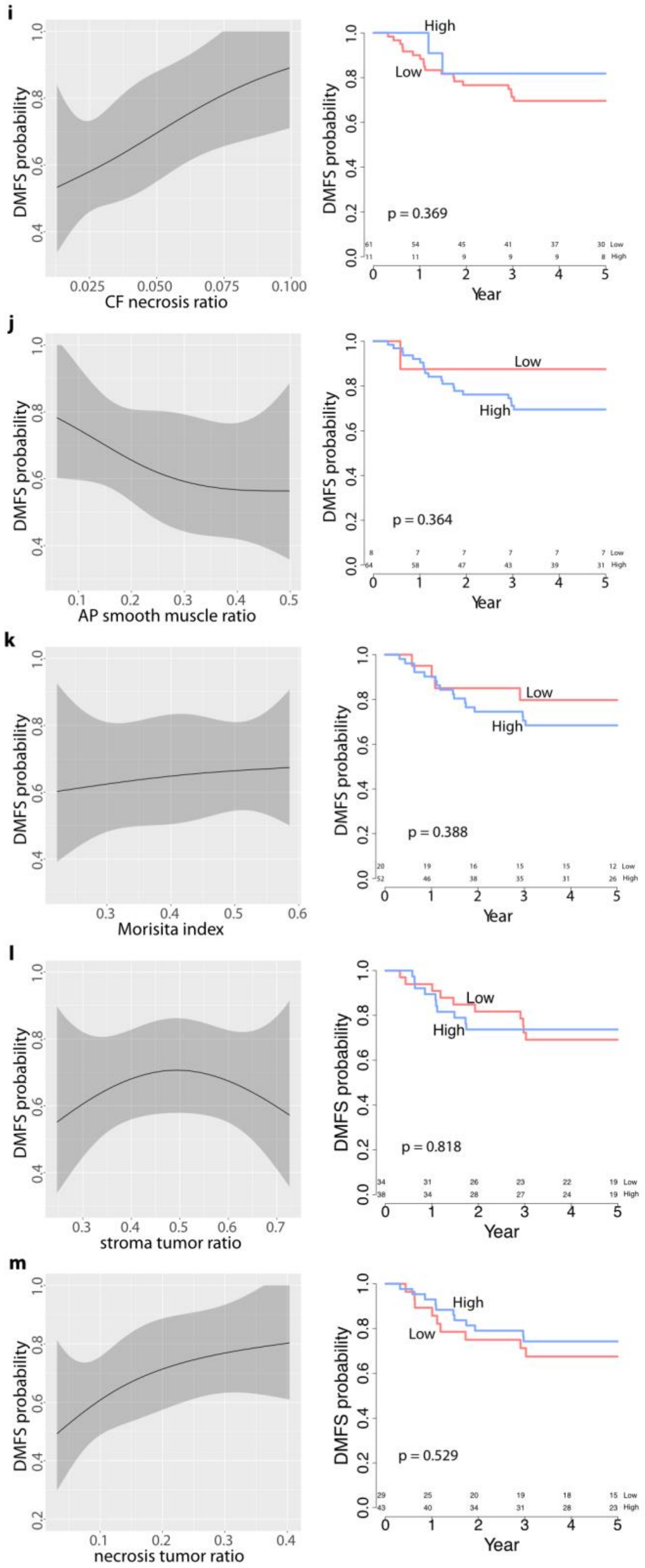

i
DMFS probability
CF necrosis ratio
High
Low
p = 0.369
Year
j
DMFS probability
AP smooth muscle ratio
Low
High
p = 0.364
Year
k
DMFS probability
Morisita index
Low
High
p = 0.388
Year
l
DMFS probability
stroma tumor ratio
Low
High
p = 0.818
Year
m
DMFS probability
necrosis tumor ratio
High
Low
p = 0.529
Year

Fig. S4. Univariate survival analysis on the UHCW cohort. Kaplan-Meier curves stratified by tumor differentiation (**a**), tumor histological type (**b**), primary tumor (T) stage (**c**), CF smooth muscle ratio (**d**, *right*), CF inflammation ratio (**e**, *right*), CF tumor-stroma interface ratio (**f**, *right*), CF tumor ratio (**g**, *right*), CF stroma ratio (**h**, *right*), CF necrosis ratio (**i**, *right*), AP smooth muscle ratio, (**j**, *right*) Morisita index (**k**, *right*), stroma-tumor ratio (**l**, *right*), and necrosis-tumor ratio (**m**, *right*). The log-rank p-value is calculated for each variable to assess if there exists a statistically significant difference between the survival distributions of different strata within the variable. The 5-year survival estimates with respect to CF smooth muscle ratio (**d**, *left*), CF inflammation ratio (**e**, *left*), CF tumor-stroma interface ratio (**f**, *left*), CF tumor ratio (**g**, *left*), CF stroma ratio (**h**, *left*), CF necrosis ratio (**i**, *left*), AP smooth muscle ratio (**j**, *left*), Morisita index (**k**, *left*), and stroma-tumor ratio (**l**, *left*), and necrosis-tumor ratio (**m**, *right*). The shaded gray areas correspond to the 95% confidence intervals of the estimates.

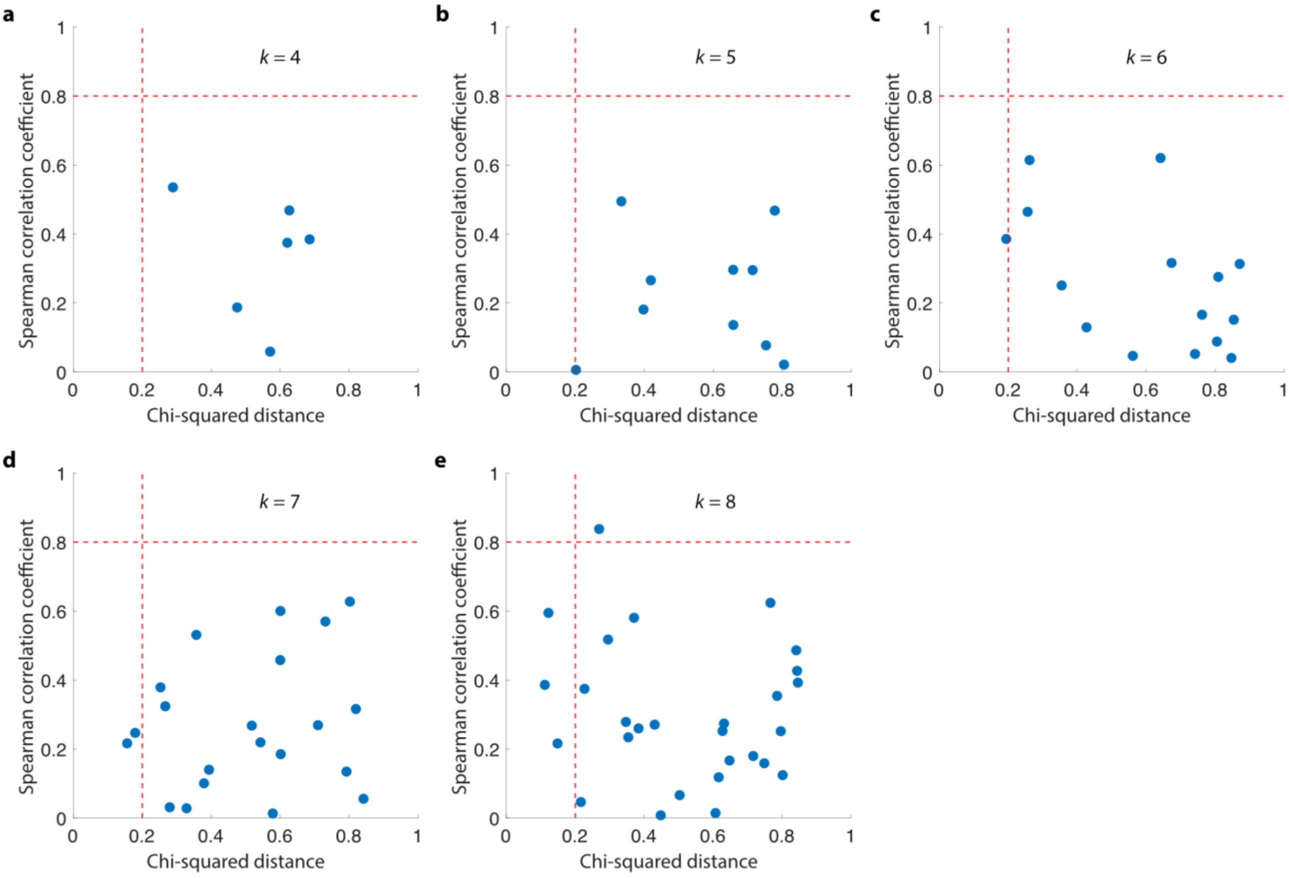

Fig. S5. Selection of the number of cell-cell connection frequency based tissue phenotypes. In determining the number of phenotypes (*k*), two criteria are taken into account: 1) similarity between phenotypes, and 2) correlation between features calculated based on the phenotypes. Each tissue phenotype should be less similar to the others and the features derived from these phenotypes should be less correlated. We measure the similarity between a pair of phenotypes in terms of a chi-squared distance between medoids that represent them. Then we calculate a feature value for each individual phenotype as the ratio of the area of that tissue phenotype to the total tissue area, and we compute the correlation between a pair of features using Spearman correlation coefficient. The chi-squared distance which is less than 0.2 (vertical red line) and the Spearman correlation coefficient which is greater than 0.8 (horizontal red line) are considered undesirable. The figures show the relationship between the chi-squared distance and the Spearman correlation coefficient of individual pairs of phenotypes with respect to k = 4 (**a**), k = 5 (**b**), k = 6 (**c**), k = 7 (**d**), and k = 8 (**e**). We select the number of phenotypes as k = 6 since it is the largest number that still retains the desirable distance and correlation for all pairs of phenotypes.

Table S1. Prognostic values of different features according to multivariate logistic regression analysis.

Feature	Feature value		Odds ratio factor			Likelihood ratio test p-value	AUC
	Baseline	Change	Estimate	Lower 95% CI	Upper 95% CI		
**Connection frequency based tissue phenotypic features**							
**CF smooth muscle ratio**	0.161	0.368	2.101	0.919	4.801	**0.019***	0.591
Differentiation	MD	PD	1.49	0.462	4.806	0.864	
Histological type	Adenocarcinoma	Mucinous	0.735	0.166	3.259	0.614	
T stage	pT3	pT4	2.631	0.866	7.991	0.074	
Cohort	UHCW	HGH	1.325	0.42	4.18	0.694	
**CF inflammation ratio**	0.042	0.139	0.305	0.11	0.846	**0.04***	0.572
Differentiation	MD	PD	1.256	0.398	3.968	0.972	
Histological type	Adenocarcinoma	Mucinous	0.846	0.2	3.586	0.767	
T stage	pT3	pT4	2.025	0.679	6.042	0.198	
Cohort	UHCW	HGH	1.292	0.404	4.133	0.705	
**CF tumor-stroma interface ratio**	0.116	0.231	0.974	0.476	1.994	0.842	0.539
Differentiation	MD	PD	1.57	0.519	4.746	0.695	
Histological type	Adenocarcinoma	Mucinous	0.781	0.183	3.329	0.685	
T stage	pT3	pT4	2.29	0.74	7.085	0.126	
Cohort	UHCW	HGH	0.883	0.316	2.469	0.755	
**CF tumor ratio**	0.079	0.23	0.487	0.227	1.044	0.117	0.581
Differentiation	MD	PD	1.358	0.425	4.338	0.986	
Histological type	Adenocarcinoma	Mucinous	0.9	0.21	3.858	0.83	
T stage	pT3	pT4	1.961	0.662	5.806	0.204	
Cohort	UHCW	HGH	0.877	0.306	2.517	0.747	
**CF stroma ratio**	0.182	0.279	0.711	0.401	1.259	0.472	0.513
Differentiation	MD	PD	1.701	0.557	5.19	0.563	
Histological type	Adenocarcinoma	Mucinous	0.807	0.19	3.433	0.729	
T stage	pT3	pT4	2.183	0.747	6.376	0.136	
Cohort	UHCW	HGH	1.047	0.363	3.018	0.981	
**CF necrosis ratio**	0.023	0.054	0.753	0.356	1.589	0.433	0.522
Differentiation	MD	PD	1.962	0.609	6.316	0.531	
Histological type	Adenocarcinoma	Mucinous	0.855	0.202	3.628	0.795	
T stage	pT3	pT4	2.093	0.716	6.118	0.142	
Cohort	UHCW	HGH	0.99	0.347	2.826	0.913	
**CF smooth muscle ratio**	0.161	0.368	2.091	0.709	6.166	0.119	0.582
**CF inflammation ratio**	0.042	0.139	0.563	0.163	1.946	0.243	
Differentiation	MD	PD	1.228	0.366	4.114	0.918	
Histological type	Adenocarcinoma	Mucinous	0.73	0.163	3.274	0.614	
T stage	pT3	pT4	2.615	0.818	8.355	0.099	
Cohort	UHCW	HGH	1.419	0.428	4.701	0.614	
**Appearance based tissue phenotypic features**							
**AP smooth muscle ratio**	0.136	0.334	2.434	1.014	5.843	0.104	0.532
Differentiation	MD	PD	1.946	0.596	6.347	0.429	
Histological type	Adenocarcinoma	Mucinous	0.597	0.133	2.685	0.452	
T stage	pT3	pT4	3.484	1.027	11.814	**0.033***	
Cohort	UHCW	HGH	1.141	0.395	3.292	0.851	
**AP inflammation ratio**	0.025	0.072	0.404	0.176	0.926	**0.033***	0.581
Differentiation	MD	PD	1.396	0.445	4.378	0.801	
Histological type	Adenocarcinoma	Mucinous	0.954	0.216	4.222	0.918	
T stage	pT3	pT4	1.897	0.622	5.785	0.246	
Cohort	UHCW	HGH	0.707	0.241	2.073	0.483	
**Other features**							
**Morisita index (*Maley*)**	0.356	0.537	1.352	0.658	2.777	0.553	0.52
Differentiation	MD	PD	1.522	0.494	4.686	0.777	
Histological type	Adenocarcinoma	Mucinous	0.776	0.181	3.318	0.692	
T stage	pT3	pT4	1.911	0.65	5.614	0.216	
Cohort	UHCW	HGH	0.866	0.309	2.423	0.73	
**Stroma-tumor ratio (*Mesker, West, Huijbers*)**	0.383	0.598	0.91	0.481	1.721	0.202	0.555
Differentiation	MD	PD	1.464	0.463	4.624	0.827	
Histological type	Adenocarcinoma	Mucinous	1.08	0.248	4.711	0.935	
T stage	pT3	pT4	2.287	0.776	6.736	0.121	
Cohort	UHCW	HGH	0.96	0.323	2.854	0.872	

**Necrosis-tumor ratio (*Pollheimer, Richards, Jayasinghe*)**	0.074	0.224	0.633	0.294	1.36	0.502	0.511
Differentiation	MD	PD	1.689	0.527	5.406	0.676	
Histological type	Adenocarcinoma	Mucinous	0.808	0.189	3.453	0.746	
T stage	pT3	pT4	1.968	0.671	5.769	0.187	
Cohort	UHCW	HGH	0.905	0.322	2.55	0.788	

* statistically significant result at the 0.05 significance level

Table S2. Prognostic values of different features according to the multivariate Cox proportional hazards regression analysis on the UHCW cohort.

Feature	Feature value		Hazard ratio factor			Wald test p-value	AUC
	Baseline	Change	Estimate	Lower 95% CI	Upper 95% CI		
Connection frequency based tissue phenotypic features							
**CF smooth muscle ratio**	0.179	0.379	2.106	0.793	5.595	**0.014***	0.586
Differentiation	MD	PD	0.999	0.342	2.916	0.999	
Histological type	Adenocarcinoma	Mucinous	0.613	0.138	2.718	0.519	
T stage	pT3	pT4	2.701	0.956	7.633	0.061	
**CF inflammation ratio**	0.037	0.103	0.415	0.183	0.945	0.099	0.558
Differentiation	MD	PD	0.961	0.329	2.809	0.942	
Histological type	Adenocarcinoma	Mucinous	0.693	0.158	3.041	0.627	
T stage	pT3	pT4	1.893	0.715	5.011	0.199	
**CF tumor-stroma interface ratio**	0.116	0.241	0.802	0.379	1.696	0.74	0.526
Differentiation	MD	PD	1.168	0.394	3.463	0.78	
Histological type	Adenocarcinoma	Mucinous	0.718	0.163	3.162	0.661	
T stage	pT3	pT4	1.942	0.719	5.249	0.191	
**CF tumor ratio**	0.079	0.222	0.502	0.233	1.083	0.119	0.583
Differentiation	MD	PD	1.214	0.39	3.782	0.738	
Histological type	Adenocarcinoma	Mucinous	0.849	0.19	3.797	0.83	
T stage	pT3	pT4	1.522	0.562	4.122	0.408	
**CF stroma ratio**	0.172	0.275	0.513	0.234	1.121	0.229	0.586
Differentiation	MD	PD	1.236	0.43	3.555	0.694	
Histological type	Adenocarcinoma	Mucinous	0.632	0.144	2.775	0.543	
T stage	pT3	pT4	2.024	0.762	5.372	0.157	
**CF necrosis ratio**	0.023	0.053	0.624	0.273	1.425	0.291	0.526
Differentiation	MD	PD	1.485	0.504	4.377	0.474	
Histological type	Adenocarcinoma	Mucinous	0.912	0.204	4.065	0.903	
T stage	pT3	pT4	2.286	0.808	6.471	0.119	
Appearance based tissue phenotypic features							
**AP smooth muscle ratio**	0.141	0.341	2.952	1.2	7.262	0.052	0.573
Differentiation	MD	PD	1.431	0.473	4.332	0.526	
Histological type	Adenocarcinoma	Mucinous	0.547	0.121	2.462	0.432	
T stage	pT3	pT4	3.131	1.093	8.968	**0.034***	
**AP inflammation ratio**	0.027	0.076	0.389	0.189	0.803	**0.004***	0.638
Differentiation	MD	PD	0.9	0.297	2.73	0.853	
Histological type	Adenocarcinoma	Mucinous	1.196	0.259	5.529	0.819	
T stage	pT3	pT4	1.629	0.592	4.483	0.345	
Other features							
**Morisita index (*Maley*)**	0.356	0.537	1.376	0.612	3.094	0.343	0.517
Differentiation	MD	PD	1.124	0.383	3.297	0.831	
Histological type	Adenocarcinoma	Mucinous	0.642	0.145	2.845	0.56	
T stage	pT3	pT4	1.627	0.61	4.339	0.331	
**Stroma-tumor ratio (*Mesker, West, Huijbers*)**	0.383	0.598	0.864	0.482	1.547	0.332	0.543
Differentiation	MD	PD	1.085	0.352	3.341	0.887	
Histological type	Adenocarcinoma	Mucinous	0.916	0.2	4.197	0.911	
T stage	pT3	pT4	2.048	0.746	5.625	0.164	
**Necrosis-tumor ratio (*Pollheimer, Richards, Jayasinghe*)**	0.074	0.224	0.657	0.289	1.494	0.465	0.515
Differentiation	MD	PD	1.275	0.412	3.949	0.673	
Histological type	Adenocarcinoma	Mucinous	0.691	0.156	3.054	0.626	
T stage	pT3	pT4	1.922	0.711	5.198	0.198	
**CF smooth muscle ratio**	0.179	0.379	2.26	0.653	7.814	0.063	0.577
**AP inflammation ratio**	0.037	0.103	0.684	0.245	1.905	0.261	
Differentiation	MD	PD	0.909	0.3	2.753	0.866	
Histological type	Adenocarcinoma	Mucinous	0.582	0.13	2.6	0.478	
T stage	pT3	pT4	2.738	0.941	7.966	0.065	

* statistically significant result at the significance level 0.05

Table S3. Technical details for the nuclear detection and classification algorithms (reproduced from Sirinukunwattana *et al.* [22]).

<table>
<tr><th></th><th>Details</th></tr>
<tr><td>Dataset</td><td>This study involves 100 H&E stained histology images of colorectal adenocarcinomas. All images have a common size of 500-by-500 pixels and were cropped from non-overlapping areas of 10 whole-slide images from 9 patients, at a resolution of 0.55 micron/pixel (equivalent to 20x optical magnification). The whole-slide images were obtained using an Omnyx VL120 scanner. The cropped areas were selected to represent a variety of tissue appearances from both normal and malignant regions of the slides. It also comprised of areas with artifacts, over-staining, and failed autofocusing, to represent outliers normally found in real scenarios.

Manual annotation of nuclei was conducted mostly by an experienced pathologist (YT) and partly by a graduate student under supervision of and validation by the same pathologist. A total number of 29,756 nuclei were marked at the center for detection purposes. Out of those, there were 22,444 nuclei that were assigned a class label, i.e., epithelial, inflammatory, fibroblast, and necrotic debris. The remaining 7,312 nuclei were unlabeled. The types of nuclei that were labeled as inflammatory, include lymphocyte plasma, neutrophil and eosinophil. Within the dataset, there are in total 7,722 epithelial, 5, 712 fibroblast, 6,971 inflammatory, and 2,039 necrotic debris.</td></tr>
<tr><td>Convolutional neural networks</td><td>Network architecture

The detailed architectures of both the nuclear detection network and the nuclear classification network are shown in the tables below. The networks consist of conventional layers, including input, convolution, non-overlapping spatial max-pooling, and fully-connected layers. The detection network, in addition, included parameter estimation and spatially constrained layers. In both networks, a rectified linear unit (ReLU) activation function is used after each convolution layer and the first two fully-connected layers (1st, 3rd, 5th and 6th layer). To avoid over-fitting, dropout is implemented in the first-two fully-connected layers (5th and 6th layer, after ReLU is applied) with a dropout rate of 0.2.

The input features were selected with respect to the task. In nucleus detection, we selected hematoxylin intensity as an input feature to the detection network for each patch. Because nucleic acids inside nuclei are stained by hematoxylin, this feature is a reasonably good representation of localization of nuclei. In classification, it is necessary to use morphology (shape, size, color, and texture) to distinguish different types of nuclei. Thus, raw RGB color intensities, which constitute the overall visual appearance of nuclei, were chosen as input features to the classification network for each patch. For both networks, we set the input patch size to since the majority of the nuclei in a dataset used in our experiments have their size within this limit. We set the output patch size for the detection network to 11-by-11 pixels. Based on this output patch size, we found that the number of nuclei contained in the training output patches is mostly less than or equal to 2. Thus, the maximum number of predicted nuclei allowed in the S1 layer of the detection network was set to 2, accordingly. For more details regarding the network architecture please refer to Sirinukunwattana et al. [22].

Detection

<table>
<tr><td>Type</td><td>Filter dimensions</td><td>Input/output dimensions</td></tr>
<tr><td>Input</td><td></td><td>27x27x1</td></tr>
<tr><td>Convolution</td><td>4x4x1x36</td><td>24x24x36</td></tr>
<tr><td>Max pooling</td><td>2x2</td><td>12x12x36</td></tr>
<tr><td>Convolution</td><td>3x3x36x48</td><td>10x10x48</td></tr>
</table>
</td></tr>
</table>

Max pooling	2x2	5x5x48
Fully-connected	5x5x48x512	1x512
Fully-connected	1x12x512x512	1x512
Spatially-constrained 1 [Ref 22]	1x1x512x3	1x3
Spatially-constrained 2 [Ref 22]		11x11

Classification

Type	Filter dimensions	Input/output dimensions
Input		27x27x1
Convolution	4x4x1x36	24x24x36
Max pooling	2x2	12x12x36
Convolution	3x3x36x48	10x10x48
Max pooling	2x2	5x5x48
Fully-connected	5x5x48x512	1x512
Fully-connected	1x12x512x512	1x512
Fully-connected	1x1x512x3	1x4

Training Data Augmentation

We arbitrary rotated patches (0, 90, 180 , 270 degree) and flipped them along vertical or horizontal axis to alleviate the rotation-variant problem of the input features. To make the classification network robust to the variability of the color distribution, which is commonly found in histology images, we also arbitrary perturbed the color distribution of the training patches. This was accomplished in HSV space, where hue (H), saturation (S), and value (V) variables were separately multiplied by random numbers $r_H \in [0.95, 1.05]$ and $r_S, r_V \in [0.9, 1.1]$, respectively. In addition, we extracted multiple patches of the same nucleus at different locations to account for location-variant that could negatively affect the classification performance of the classification network. This over-sampling also allowed us to account for the class unbalance problem inherent in the dataset.

Initialization and Training of the Networks

We initialized all weights with 0 mean and 10^{-2} standard deviation Gaussian random numbers. All biases were set to 0. The networks were trained using stochastic gradient descent with momentum 0.9 and weight decay $5x10^{-4}$ for 120 epochs. We annealed the learning rate, starting from 10^{-2} for the first 60 epochs, then 10^{-3} for the next 40 epochs, and 10^{-4} for the last 20 epochs. We used 20% of the training data for validation. The optimal networks for detection and classification were selected based on the root mean square error and the classification error on the validation set, respectively.

Evaluation

Nuclear detection

The objective of this experiment is to detect all nuclei in an image by locating their center positions, regardless of their class labels.

Precision, Recall, and F1 score were used to quantitatively assess the detection performance. Here, we define the region within a radius of 6 pixels from the annotated center of each nucleus as its ground truth. If there are multiple detected points within the same ground truth region, only the one closest to the annotated center is considered as a true positive. Statistics, including the median, 1st quartile, and 3rd quartile were also calculated. This is to summarize the positively skewed distribution of the Euclidean distance between the detected points and their nearest

ground truth centers. We employed 2-fold cross-validation (50 images/fold) in the experiment.

Precision	Recall	F1 score	Median distance (Q1, Q3)
0.781	0.823	0.802	2.236 (1.414, 5)

Nuclear classification

The setting of this experiment is to classify patches of size 27-by-27 pixels, containing a nucleus at the center, into 4 classes: epithelial, inflammatory, fibroblast, and miscellaneous.

We calculated the F1 score for each class of nuclei and their average weighted by the number of nucleus samples to summarize the overall classification performance. We also considered an area under the receiver operating characteristic curve for multiclass classification (multiclass AUC) [59]. Multiclass AUC measures the probability that given a pair of samples with different class labels, a classifier will assign a high prediction score for class, say c, to the sample from class c, as compared to the sample from the other class. Here, the prediction score is given by the softmax function.

Weighted average F1 score	Multiclass AUC
0.784	0.917

Running time

In our experiment, a whole-slide image is first divided into small tiles of size 1,000-by-1,000 pixels. On a single 2.5 GHz CPU, the average execution time on an individual image tile is 47.6 s (preprocessing 27.8 s, detection 18.4 s, and classification 1.4 s). For a given whole-slide image captured at 20x optical magnification and consisting of 60,000-by-50,000 pixel dimensions, there are 750 tiles of size 1,000-by-1,000 pixels to be processed assuming only 25% of the slide contains tissue. Theoretically speaking, by using a 12–core processor machine, the average execution time of the proposed detection and classification framework is around 50 mins per slide. However, it should be noted that the execution time reported here is for a research-grade implementation of the framework which has not yet been fully optimized to increase time efficiency, nor did we employ the computational power of GPUs which can significantly speed up the execution time.
