# Supplementary material for: Novel digital signatures of tissue phenotypes for predicting distant metastasis in colorectal cancer
Source: Sci Rep. 2018 Sep 12;8:13692. doi: 10.1038/s41598-018-31799-3 (PMC6135776; doi:10.1038/s41598-018-31799-3)
Supplement: Supplementary file 2 — Supplementary materials: R codes [file 41598_2018_31799_MOESM2_ESM.pdf]

Novel digital signatures of tissue phenotypes for predicting distant metastasis in colorectal cancer: R code for statistical analyses

Korsuk Sirinukunwattana, David Snead, David Epstein, Zia Aftab, Imaad Mujeeb, Yee Wah Tsang, Ian Cree, and Nasir Rajpoot

Contents

Multiple Imputation **1**

Association Between Phenotypic and Clinical Features **2**

Univariate Logistic Regression Analysis **3**

Multivariate Logistic Regression Analysis **8**

Univariate Cox Proportional Hazards Regression Analysis **13**

Multivariate Cox Proportional Hazards Regression Analysis **18**

Multiple Imputation

```
# Load libraries
library(survival)
library(mice)
library(rms)

# Clear the working space
rm(list = ls())

# Load the data
load("data.RData")

# Perform multiple imputation for missing data.  The missing
# features are(1) differentation (2) histological type.

# Initialisation
ini <- mice(data, m = 5, maxit = 0, seed = 7887)
pred <- ini$predictorMatrix

# Do not use metastasis status, survival time, or features
# calculated from images for imputation
pred[, "met.status"] <- 0
pred[, "time"] <- 0
pred[, "event"] <- 0
pred[, 7:dim(data)[2]] <- 0

# Also no imputation on time and event for Qatari cases
```

```r
pred["time", ] <- 0
pred["event", ] <- 0

# Generate 100 datasets of imputed dataset
imp <- mice(data, m = 100, maxit = 20, pred = pred, pri = TRUE,
    seed = 7887)

# Save
save(imp, file = "multiple.imputation.RData")
```

Association Between Phenotypic and Clinical Features

```r
# Load libraries
library(survival)
library(mice)
library(rms)

# Clear the working space
rm(list = ls())

# Set a seed for a random number generator for
# reproducibility
load("multiple.imputation.RData")

# Phenotypic features
feat.x = c("CF.smooth.muscle.ratio", "CF.inflammation.ratio",
    "CF.tumor.stroma.interface.ratio", "CF.tumor.ratio", "CF.stroma.ratio",
    "CF.necrosis.ratio")

# Clinical features
feat.y = c("differentiation", "histological.type", "T.stage")

# Calculate p-value and r-squared of Mann-Whitney test
pval.matrix <- array(0, dim = c(3, length(feat.x), 100))
r.squared.matrix <- array(0, dim = c(3, length(feat.x), 100))

for (i.impute in c(1:100)) {
    all.features <- complete(imp, i.impute)

    # Remove the time and event variables of Qatari cases
    all.features$time[c(79:length(all.features$time))] <- NA
    all.features$event[c(79:length(all.features$time))] <- NA

    # Remove cases with met at diagnosis
    all.features <- all.features[all.features$time != 0 | is.na(all.features$time),
        ]

    for (i.x in c(1:length(feat.x))) {
        for (i.y in c(1:length(feat.y))) {
            x <- as.numeric(unlist(all.features[feat.x[i.x]]))
            y <- as.numeric(unlist(all.features[feat.y[i.y]]))
```

```
            pval.matrix[i.y, i.x, i.impute] <- wilcox.test(x ~
                factor(y))$p.value
            n <- length(y)
            p.value <- wilcox.test(x ~ factor(y))$p.value
            r.squared.matrix[i.y, i.x, i.impute] <- qnorm(p.value/2)^2/n
        }
    }
}

# Calculate the median across imputation datasets
pval <- t(apply(pval.matrix, c(1, 2), median))
r.squared <- t(apply(r.squared.matrix, c(1, 2), median))

# Output p-value table
colnames(pval) <- feat.y
rownames(pval) <- feat.x

# Output r-squared table
colnames(r.squared) <- feat.y
rownames(r.squared) <- feat.x

# Display results
round(pval, 3)
round(r.squared, 3)
```

Univariate Logistic Regression Analysis

```
# Load libraries
library(survival)
library(mice)
library(rms)

# Clear the working space
rm(list = ls())

# Define a function to merge results from different multiple
# imputation datasets
merge.result <- function(onefeat) {
    call <- match.call()
    using.Design <- FALSE
    stats.ok2average <- c("linear.predictors", "fitted.values",
        "stats", "means", "icoef", "scale", "center", "y.imputed")
    n.impute <- length(onefeat)
    for (i in 1:n.impute) {
        f <- onefeat[[i]]

        cof <- f$coef
        v <- do.call("vcov", c(list(object = f, intercepts = "all"),
            NULL))
        if (i == 1) {
            assign <- f$assign
```

```r
            ns <- num.intercepts(f)
            ik <- coef.intercepts <- NULL
            if (ns > 0) {
                ik <- attr(v, "intercepts")
                if (length(ik)) {
                  if (ik == "all")
                    ik <- 1:ns else if (ik == "none")
                    ik <- 0
                  lenik <- length(ik)
                  if (length(ik) == 1 && ik == 0)
                    lenik <- 0
                  if (lenik != ns) {
                    for (j in 1:length(assign)) assign[[j]] <- assign[[j]] -
                      (ns - lenik)
                    coef.intercepts <- ik
                  }
                }
            }
        }
        if (length(ik))
            cof <- c(cof[ik], cof[-(1:ns)])
        nvar0 <- length(cof)
        nvar <- nrow(v)
        if (nvar > nvar0) {
            cof <- c(cof, log(f$scale))
            names(cof) <- c(names(f$coef), if ((nvar - nvar0) ==
                1) "Log(scale)" else names(f$scale))
        }
        if (i == 1) {
            vavg <- 0 * v
            p <- length(cof)
            bar <- rep(0, p)
            vname <- names(cof)
            cov <- matrix(0, nrow = p, ncol = p, dimnames = list(vname,
                vname))
            astats <- NULL
            fitcomp <- names(f)[names(f) %in% stats.ok2average]
            if (length(fitcomp))
                for (ncomp in fitcomp) astats[[ncomp]] <- f[[ncomp]]
            if (inherits(f, "Design") | inherits(f, "rms")) {
                using.Design <- TRUE
                da <- f$Design
            }
        }
        vavg <- vavg + v
        bar <- bar + cof
        cof <- as.matrix(cof)
        cov <- cov + cof %*% t(cof)
        if (i > 1 && length(fitcomp))
            for (ncomp in fitcomp) astats[[ncomp]] <- astats[[ncomp]] +
                f[[ncomp]]
    }
```

```
    vavg <- vavg/n.impute
    bar <- bar/n.impute
    bar <- as.matrix(bar)
    cov <- (cov - n.impute * bar %*% t(bar))/(n.impute - 1)
    U <- diag(vavg)
    B <- diag(cov)
    cov <- vavg + (n.impute + 1)/n.impute * cov
    r <- diag(cov)/diag(vavg)
    names(r) <- vname
    tau <- (1 + 1/n.impute) * B/U
    missingInfo <- tau/(1 + tau)
    dfmi <- (n.impute - 1) * ((1 + 1/tau)^2)
    if (length(fitcomp))
        for (ncomp in fitcomp) f[[ncomp]] <- astats[[ncomp]]/n.impute
    if (TRUE) {
        cat("\nVariance Inflation Factors Due to Imputation:\n\n")
        print(round(r, 2))
        cat("\nRate of Missing Information:\n\n")
        print(round(missingInfo, 2))
        cat("\nd.f. for t-distribution for Tests of Single Coefficients:\n\n")
        print(round(dfmi, 2))
        if (length(fitcomp)) {
            cat("\nThe following fit components were averaged over the",
                n.impute, "model fits:\n\n")
            cat(" ", fitcomp, "\n\n")
        }
    }
    f$coefficients <- drop(bar)
    if (length(coef.intercepts))
        attr(f$coefficients, "intercepts") <- coef.intercepts
    attr(cov, "intercepts") <- ik
    f$var <- cov
    f$variance.inflation.impute <- r
    f$missingInfo <- missingInfo
    f$dfmi <- dfmi
    f$formula <- formula
    f$assign <- assign
    f$call <- call
    if (using.Design)
        options(Design.attr = NULL)
    class(f) <- c("fit.mult.impute", class(f))
    f
}

# Load the multiple imputation data
load("multiple.imputation.RData")

# Set a seed for a random number generator for
# reproducibility
set.seed(7887)

# Features.  Use restricted cubic spline (rcs) to estimate
# interpolate shapes of continuous variables.
```

```r
feat <- c("differentiation", "histological.type", "T.stage",
    "cohort", "rcs(CF.smooth.muscle.ratio,3)", "rcs(CF.inflammation.ratio,3)",
    "rcs(CF.tumor.stroma.interface.ratio,3)", "rcs(CF.tumor.ratio,3)",
    "rcs(CF.stroma.ratio,3)", "rcs(CF.necrosis.ratio,3)", "rcs(AP.smooth.muscle.ratio,3)",
    "rcs(AP.inflammation.ratio,3)", "rcs(Morisita.index,3)",
    "rcs(stroma.tumor.ratio,3)", "rcs(necrosis.tumor.ratio,3)")

# Perform univariate logistic regression
OR.factor <- vector("list", length(feat))  # odds ratio factor
lower.95CI <- vector("list", length(feat))  # 95% lower bound of odds ratio factor
upper.95CI <- vector("list", length(feat))  # 95% upper bound of odds ratio factor

low <- vector("list", length(feat))  # 1st quartile
high <- vector("list", length(feat))  # 3rd quartile

pval <- vector("list", length(feat))  # likelihood ratio p-value
c = vector("list", length(feat))  # concordance (AUC)

for (ifeat in c(1:length(feat))) {
    onefeat <- vector("list", 100)

    for (i.impute in c(1:100)) {
        # Load a single instance of the multiple imputation datasets
        all.features <- complete(imp, i.impute)

        # Add cohort indicator
        all.features$cohort <- c(rep(0, 78), rep(1, 30))

        # Remove the time and event variables of Qatari cases
        all.features$time[c(79:length(all.features$time))] <- NA
        all.features$event[c(79:length(all.features$time))] <- NA

        # Remove cases with met at diagnosis
        all.features <- all.features[all.features$time != 0 |
            is.na(all.features$time), ]

        all.features$met.status <- factor(all.features$met.status)
        all.features$histological.type <- factor(all.features$histological.type)
        all.features$differentiation <- factor(all.features$differentiation)
        all.features$T.stage <- factor(all.features$T.stage)
        all.features$cohort <- factor(all.features$cohort)

        dd <- datadist(all.features)
        options(datadist = "dd", contrasts = c("contr.treatment",
            "contr.treatment"))

        formu <- paste("met.status~", paste(feat[ifeat], collapse = "+"))  # formula
        onefeat[[i.impute]] <- lrm(as.formula(formu), x = TRUE,
            y = TRUE, data = all.features)  # fit logistic regression model
        print(i.impute)
    }  # Loop throught different imputations

    model <- merge.result(onefeat)  # Merge model
```

```r
    my.valid <- validate(model, method = "boot", B = 200)  # validate the model on 1000 bootstraping. i
    c[[ifeat]] <- (1 + my.valid[1, 5])/2

    if (feat[ifeat] == "differentiation") {
        OR.factor[[ifeat]] <- summary(model, differentiation = "MD")[seq(2,
            dim(summary(model))[1], 2), 4]
        lower.95CI[[ifeat]] <- summary(model, differentiation = "MD")[seq(2,
            dim(summary(model))[1], 2), 6]
        upper.95CI[[ifeat]] <- summary(model, differentiation = "MD")[seq(2,
            dim(summary(model))[1], 2), 7]

        low[[ifeat]] <- summary(model, differentiation = "MD")[seq(2,
            dim(summary(model))[1], 2), 1]
        high[[ifeat]] <- summary(model, differentiation = "MD")[seq(2,
            dim(summary(model))[1], 2), 2]
    } else {
        OR.factor[[ifeat]] <- summary(model)[seq(2, dim(summary(model))[1],
            2), 4]
        lower.95CI[[ifeat]] <- summary(model)[seq(2, dim(summary(model))[1],
            2), 6]
        upper.95CI[[ifeat]] <- summary(model)[seq(2, dim(summary(model))[1],
            2), 7]

        low[[ifeat]] <- summary(model)[seq(2, dim(summary(model))[1],
            2), 1]
        high[[ifeat]] <- summary(model)[seq(2, dim(summary(model))[1],
            2), 2]
    }

    pval[[ifeat]] <- model$stats["P"]
}  # Loop through features


# Output table
output.table <- round(data.frame(unlist(low), unlist(high), unlist(OR.factor),
    unlist(lower.95CI), unlist(upper.95CI), unlist(pval), unlist(c)),
    3)
colnames(output.table) <- c("Q1", "Q3", "OR.factor", "lower.95CI",
    "upper.95CI", "p.val", "AUC")
rownames(output.table) <- c("differentiation", "histological.type",
    "T.stage", "cohort", "CF.smooth.muscle.ratio", "CF.inflammation.ratio",
    "CF.tumor.stroma.interface.ratio", "CF.tumor.ratio", "CF.stroma.ratio",
    "CF.necrosis.ratio", "AP.smooth.muscle.ratio", "AP.inflammation.ratio",
    "Morisita.index", "stroma.tumor.ratio", "necrosis.tumor.ratio")

# Display results
round(output.table, 3)
```

Multivariate Logistic Regression Analysis

```
# Load libraries
library(survival)
library(mice)
library(rms)

# Clear working space
rm(list = ls())

# Define a function to merge results from multiple imputation
# dataset
merge.result <- function(onefeat) {
    call <- match.call()
    using.Design <- FALSE
    stats.ok2average <- c("linear.predictors", "fitted.values",
        "stats", "means", "icoef", "scale", "center", "y.imputed")
    n.impute <- length(onefeat)
    for (i in 1:n.impute) {
        f <- onefeat[[i]]

        cof <- f$coef
        v <- do.call("vcov", c(list(object = f, intercepts = "all"),
            NULL))
        if (i == 1) {
            assign <- f$assign
            ns <- num.intercepts(f)
            ik <- coef.intercepts <- NULL
            if (ns > 0) {
                ik <- attr(v, "intercepts")
                if (length(ik)) {
                  if (ik == "all")
                    ik <- 1:ns else if (ik == "none")
                    ik <- 0
                  lenik <- length(ik)
                  if (length(ik) == 1 && ik == 0)
                    lenik <- 0
                  if (lenik != ns) {
                    for (j in 1:length(assign)) assign[[j]] <- assign[[j]] -
                      (ns - lenik)
                    coef.intercepts <- ik
                  }
                }
            }
        }
        if (length(ik))
            cof <- c(cof[ik], cof[-(1:ns)])
        nvar0 <- length(cof)
        nvar <- nrow(v)
        if (nvar > nvar0) {
            cof <- c(cof, log(f$scale))
            names(cof) <- c(names(f$coef), if ((nvar - nvar0) ==
                1) "Log(scale)" else names(f$scale))
```

```r
    }
    if (i == 1) {
        vavg <- 0 * v
        p <- length(cof)
        bar <- rep(0, p)
        vname <- names(cof)
        cov <- matrix(0, nrow = p, ncol = p, dimnames = list(vname,
            vname))
        astats <- NULL
        fitcomp <- names(f)[names(f) %in% stats.ok2average]
        if (length(fitcomp))
            for (ncomp in fitcomp) astats[[ncomp]] <- f[[ncomp]]
        if (inherits(f, "Design") | inherits(f, "rms")) {
            using.Design <- TRUE
            da <- f$Design
        }
    }
    vavg <- vavg + v
    bar <- bar + cof
    cof <- as.matrix(cof)
    cov <- cov + cof %*% t(cof)
    if (i > 1 && length(fitcomp))
        for (ncomp in fitcomp) astats[[ncomp]] <- astats[[ncomp]] +
            f[[ncomp]]
}

vavg <- vavg/n.impute
bar <- bar/n.impute
bar <- as.matrix(bar)
cov <- (cov - n.impute * bar %*% t(bar))/(n.impute - 1)
U <- diag(vavg)
B <- diag(cov)
cov <- vavg + (n.impute + 1)/n.impute * cov
r <- diag(cov)/diag(vavg)
names(r) <- vname
tau <- (1 + 1/n.impute) * B/U
missingInfo <- tau/(1 + tau)
dfmi <- (n.impute - 1) * ((1 + 1/tau)^2)
if (length(fitcomp))
    for (ncomp in fitcomp) f[[ncomp]] <- astats[[ncomp]]/n.impute
if (TRUE) {
    cat("\nVariance Inflation Factors Due to Imputation:\n\n")
    print(round(r, 2))
    cat("\nRate of Missing Information:\n\n")
    print(round(missingInfo, 2))
    cat("\nd.f. for t-distribution for Tests of Single Coefficients:\n\n")
    print(round(dfmi, 2))
    if (length(fitcomp)) {
        cat("\nThe following fit components were averaged over the",
            n.impute, "model fits:\n\n")
        cat(" ", fitcomp, "\n\n")
    }
}
```

```r
    f$coefficients <- drop(bar)
    if (length(coef.intercepts))
        attr(f$coefficients, "intercepts") <- coef.intercepts
    attr(cov, "intercepts") <- ik
    f$var <- cov
    f$variance.inflation.impute <- r
    f$missingInfo <- missingInfo
    f$dfmi <- dfmi
    f$formula <- formula
    f$assign <- assign
    f$call <- call
    if (using.Design)
        options(Design.attr = NULL)
    class(f) <- c("fit.mult.impute", class(f))
    f
}

# Load the multiple imputation data
load("multiple.imputation.RData")

# Set a seed for a random number generator for
# reproducibility
set.seed(7887)

# Select features. Use restricted cubic spline (rcs) to
# estimate interpolate shapes of continuous variables.

# Automatically calculated features
feat <- c("rcs(CF.smooth.muscle.ratio,3)", "rcs(CF.inflammation.ratio,3)",
    "rcs(CF.tumor.stroma.interface.ratio,3)", "rcs(CF.tumor.ratio,3)",
    "rcs(CF.stroma.ratio,3)", "rcs(CF.necrosis.ratio,3)", "rcs(CF.smooth.muscle.ratio,3) + rcs(CF.infla
    "rcs(AP.smooth.muscle.ratio,3)", "rcs(AP.inflammation.ratio,3)",
    "rcs(Morisita.index,3)", "rcs(stroma.tumor.ratio,3)", "rcs(necrosis.tumor.ratio,3)")

# Clinical features
feat2 <- c("differentiation", "histological.type", "T.stage",
    "cohort")

# Perform multivariate logistic regression
for (ifeat in c(1:length(feat))) {
    onefeat <- vector("list", 100)
    for (i.impute in c(1:100)) {
        # Load an instance of the multiple imputation datasets
        all.features <- complete(imp, i.impute)

        # Add cohort indicator
        all.features$cohort <- c(rep(0, 78), rep(1, 30))

        # Remove time and event variables of Qatari cases
        all.features$time[c(79:length(all.features$time))] <- NA
        all.features$event[c(79:length(all.features$time))] <- NA

        # Remove cases with met at diagnosis
```

```r
    all.features <- all.features[all.features$time != 0 |
        is.na(all.features$time), ]

    all.features$met.status <- factor(all.features$met.status)
    all.features$histological.type <- factor(all.features$histological.type)
    all.features$differentiation <- factor(all.features$differentiation)
    all.features$T.stage <- factor(all.features$T.stage)
    all.features$cohort <- factor(all.features$cohort)

    dd <- datadist(all.features)
    options(datadist = "dd", contrasts = c("contr.treatment",
        "contr.treatment"))

    # Formula
    formu <- paste("met.status~", paste(c(feat[ifeat], feat2),
        collapse = "+"))

    # Fit a logistic regression model
    onefeat[[i.impute]] <- tryCatch(lrm(as.formula(formu),
        x = TRUE, y = TRUE, data = all.features), error = function(e) {
        NULL
    }, warning = function(w) {
        NULL
    })

    print(i.impute)
}  # Loop throught different imputation dataset

# Remove null model
onefeat <- onefeat[!sapply(onefeat, is.null)]

# Merge model
model.full <- merge.result(onefeat)

# Validate
my.valid <- validate(model.full, method = "boot", B = 1000)
c <- (1 + my.valid[1, 5])/2

# OR factor
OR.factor <- summary(model.full, differentiation = "MD")[seq(2,
    dim(summary(model.full))[1], 2), 4]
names(OR.factor) <- rownames(summary(model.full, differentiation = "MD"))[seq(1,
    dim(summary(model.full))[1], 2)]

# Lower 95% CI
lower.95CI <- summary(model.full, differentiation = "MD")[seq(2,
    dim(summary(model.full))[1], 2), 6]
names(lower.95CI) <- rownames(summary(model.full, differentiation = "MD"))[seq(1,
    dim(summary(model.full))[1], 2)]

# Upper 95% CI
upper.95CI <- summary(model.full, differentiation = "MD")[seq(2,
    dim(summary(model.full))[1], 2), 7]
```

```r
names(upper.95CI) <- rownames(summary(model.full, differentiation = "MD"))[seq(1,
    dim(summary(model.full))[1], 2)]

# Perform likelihood ratio test for each feature
feat3 <- c(feat[ifeat], feat2)
pos = regexpr("\\+", feat3[1])
if (pos > 0) {
    feat3 <- c(strsplit(feat3[1], "\\+")[[1]], feat3[-1])
}
# likelihood ratio p-value
pval <- vector("list", length(feat3))
for (jfeat in c(1:length(feat3))) {

    onefeat <- vector("list", 100)
    for (i.impute in c(1:100)) {
        # Load multiple imputation dataset
        all.features <- complete(imp, i.impute)

        # add cohort indicator
        all.features$cohort <- c(rep(0, 78), rep(1, 30))

        # Remove time and event for Qatari cases
        all.features$time[c(79:length(all.features$time))] <- NA
        all.features$event[c(79:length(all.features$time))] <- NA

        # Remove cases with met at diagnosis
        all.features <- all.features[all.features$time !=
            0 | is.na(all.features$time), ]

        all.features$met.status <- factor(all.features$met.status)
        all.features$histological.type <- factor(all.features$histological.type)
        all.features$T.stage <- factor(all.features$T.stage)
        all.features$differentiation <- factor(all.features$differentiation)
        all.features$cohort <- factor(all.features$cohort)

        dd <- datadist(all.features)
        options(datadist = "dd")
        options(contrasts = c("contr.treatment", "contr.treatment"))

        # Formula
        formu <- paste("met.status~", paste(feat3[-jfeat],
            collapse = "+"))

        # Fit a logistic regression model
        onefeat[[i.impute]] <- tryCatch(lrm(as.formula(formu),
            x = TRUE, y = TRUE, data = all.features), error = function(e) {
            NULL
        }, warning = function(w) {
            NULL
        })
        print(i.impute)
    }  # fit model exclude jfeat
    onefeat <- onefeat[!sapply(onefeat, is.null)]  # remove null model
```

```
        model.reduce <- merge.result(onefeat)  # merge model

        # Calculate a log-likelihood ratio p-value
        pval[[jfeat]] <- 1 - pchisq(model.reduce$deviance[2] -
            model.full$deviance[2], df = length(model.full$coefficients) -
            length(model.reduce$coefficients))
    }
    pval <- unlist(pval)

    # Output table
    output.table <- round(data.frame(OR.factor, lower.95CI, upper.95CI,
        pval, c), 3)

    # Display results
    output.table
}
```

Univariate Cox Proportional Hazards Regression Analysis

```
# Load libraries
library(survival)
library(mice)
library(rms)

# Clear the working space
rm(list = ls())

# Define a function to merge results from multiple imputation
# dataset
merge.result <- function(onefeat) {
    call <- match.call()
    using.Design <- FALSE
    stats.ok2average <- c("linear.predictors", "fitted.values",
        "stats", "means", "icoef", "scale", "center", "y.imputed")
    n.impute <- length(onefeat)
    for (i in 1:n.impute) {
        f <- onefeat[[i]]

        cof <- f$coef
        v <- do.call("vcov", c(list(object = f, intercepts = "all"),
            NULL))
        if (i == 1) {
            assign <- f$assign
            ns <- num.intercepts(f)
            ik <- coef.intercepts <- NULL
            if (ns > 0) {
                ik <- attr(v, "intercepts")
                if (length(ik)) {
                  if (ik == "all")
                    ik <- 1:ns else if (ik == "none")
                    ik <- 0
```

```
                lenik <- length(ik)
                if (length(ik) == 1 && ik == 0)
                  lenik <- 0
                if (lenik != ns) {
                  for (j in 1:length(assign)) assign[[j]] <- assign[[j]] -
                    (ns - lenik)
                  coef.intercepts <- ik
                }
              }
          }
      }
      if (length(ik))
          cof <- c(cof[ik], cof[-(1:ns)])
      nvar0 <- length(cof)
      nvar <- nrow(v)
      if (nvar > nvar0) {
          cof <- c(cof, log(f$scale))
          names(cof) <- c(names(f$coef), if ((nvar - nvar0) ==
              1) "Log(scale)" else names(f$scale))
      }
      if (i == 1) {
          vavg <- 0 * v
          p <- length(cof)
          bar <- rep(0, p)
          vname <- names(cof)
          cov <- matrix(0, nrow = p, ncol = p, dimnames = list(vname,
              vname))
          astats <- NULL
          fitcomp <- names(f)[names(f) %in% stats.ok2average]
          if (length(fitcomp))
              for (ncomp in fitcomp) astats[[ncomp]] <- f[[ncomp]]
          if (inherits(f, "Design") | inherits(f, "rms")) {
              using.Design <- TRUE
              da <- f$Design
          }
      }
      vavg <- vavg + v
      bar <- bar + cof
      cof <- as.matrix(cof)
      cov <- cov + cof %*% t(cof)
      if (i > 1 && length(fitcomp))
          for (ncomp in fitcomp) astats[[ncomp]] <- astats[[ncomp]] +
              f[[ncomp]]
  }

  vavg <- vavg/n.impute
  bar <- bar/n.impute
  bar <- as.matrix(bar)
  cov <- (cov - n.impute * bar %*% t(bar))/(n.impute - 1)
  U <- diag(vavg)
  B <- diag(cov)
  cov <- vavg + (n.impute + 1)/n.impute * cov
  r <- diag(cov)/diag(vavg)
```

```r
    names(r) <- vname
    tau <- (1 + 1/n.impute) * B/U
    missingInfo <- tau/(1 + tau)
    dfmi <- (n.impute - 1) * ((1 + 1/tau)^2)
    if (length(fitcomp))
        for (ncomp in fitcomp) f[[ncomp]] <- astats[[ncomp]]/n.impute
    if (TRUE) {
        cat("\nVariance Inflation Factors Due to Imputation:\n\n")
        print(round(r, 2))
        cat("\nRate of Missing Information:\n\n")
        print(round(missingInfo, 2))
        cat("\nd.f. for t-distribution for Tests of Single Coefficients:\n\n")
        print(round(dfmi, 2))
        if (length(fitcomp)) {
            cat("\nThe following fit components were averaged over the",
                n.impute, "model fits:\n\n")
            cat(" ", fitcomp, "\n\n")
        }
    }
    f$coefficients <- drop(bar)
    if (length(coef.intercepts))
        attr(f$coefficients, "intercepts") <- coef.intercepts
    attr(cov, "intercepts") <- ik
    f$var <- cov
    f$variance.inflation.impute <- r
    f$missingInfo <- missingInfo
    f$dfmi <- dfmi
    f$formula <- formula
    f$assign <- assign
    f$call <- call
    if (using.Design)
        options(Design.attr = NULL)
    class(f) <- c("fit.mult.impute", class(f))
    f
}

# Define a function to calculate a corrected log-rank p-value
corrected.pval <- function(feature, time, event) {
    low <- quantile(feature, 0.1)
    up <- quantile(feature, 0.9)
    thresh <- seq(low, up, by = 0.005)

    p.vector <- vector("numeric", length(thresh))
    for (j in c(1:length(thresh))) {
        cut <- ifelse(feature > thresh[j], 1, 0)
        test <- survdiff(formula = Surv(time, event) ~ cut)
        p.val <- 1 - pchisq(test$chisq, length(test$n) - 1)
        p.vector[j] <- p.val
    }

    p.min <- min(p.vector)
    z <- qnorm(1 - p.min/2)
    p.corr <- dnorm(z) * (z - 1/z) * log(9^2) + 4 * dnorm(z)/z
```

```
}

# Set a seed for a random number generator for
# reproducibility
set.seed(7887)

# Load the multiple imputation data
load("multiple.imputation.RData")

# Select features. Use restricted cubic spline (rcs) to
# estimate interpolate shapes of continuous variables.
feat <- c("differentiation", "histological.type", "T.stage",
    "rcs(CF.smooth.muscle.ratio,3)", "rcs(CF.inflammation.ratio,3)",
    "rcs(CF.tumor.stroma.interface.ratio,3)", "rcs(CF.tumor.ratio,3)",
    "rcs(CF.stroma.ratio,3)", "rcs(CF.necrosis.ratio,3)", "rcs(AP.smooth.muscle.ratio,3)",
    "rcs(AP.inflammation.ratio,3)", "rcs(Morisita.index,3)",
    "rcs(stroma.tumor.ratio,3)", "rcs(necrosis.tumor.ratio,3)")

feat.name <- c("differentiation", "histological.type", "T.stage",
    "CF.smooth.muscle.ratio", "CF.inflammation.ratio", "CF.tumor.stroma.interface.ratio",
    "CF.tumor.ratio", "CF.stroma.ratio", "CF.necrosis.ratio",
    "AP.smooth.muscle.ratio", "AP.inflammation.ratio", "Morisita.index",
    "stroma.tumor.ratio", "necrosis.tumor.ratio")

# Predefine variables
HR.factor <- vector("list", length(feat))
lower.95CI <- vector("list", length(feat))
upper.95CI <- vector("list", length(feat))
pval <- vector("list", length(feat))
corrected.log.rank.pval <- vector("list", length(feat))

c <- vector("list", length(feat))

low <- vector("list", length(feat))
high <- vector("list", length(feat))

for (ifeat in c(1:length(feat))) {
    onefeat <- vector("list", 100)
    for (i.impute in c(1:100)) {
        # Load an instance of the multiple imputation datasets
        all.features <- complete(imp, i.impute)

        # Remove the Qatari cases
        all.features <- all.features[c(1:78), ]

        # Remove cases with met at diagnosis
        all.features <- all.features[all.features$time != 0 |
            is.na(all.features$time), ]

        all.features$histological.type <- factor(all.features$histological.type)
        all.features$T.stage <- factor(all.features$T.stage)
        all.features$differentiation <- factor(all.features$differentiation)
        all.features$met.status <- factor(all.features$met.status)
```

```r
    # Survival data
    units(all.features$time) <- "Year"  # set time unit

    # Set data distribution for cph
    dd <- datadist(all.features)
    options(datadist = "dd", contrasts = c("contr.treatment",
        "contr.treatment"))

    # Fit a univariate cox proportional harzards regression
    formu <- paste("Surv(time,event)~", feat[ifeat], sep = "")
    onefeat[[i.impute]] <- tryCatch(cph(as.formula(formu),
        data = all.features, x = T, y = T, surv = T, time.inc = 0.1),
        error = function(e) {
            NULL
        }, warning = function(w) {
            NULL
        })  # fit logistic regression
}  # Loop through all multiple imputation dataset
onefeat <- onefeat[!sapply(onefeat, is.null)]  # remove null model
model <- merge.result(onefeat)  # merge model
model$formula <- NULL

if (feat[ifeat] == "differentiation") {
    HR.factor[[ifeat]] <- summary(model, differentiation = "MD")[seq(2,
        dim(summary(model))[1], 2), 4]
    lower.95CI[[ifeat]] <- summary(model, differentiation = "MD")[seq(2,
        dim(summary(model))[1], 2), 6]
    upper.95CI[[ifeat]] <- summary(model, differentiation = "MD")[seq(2,
        dim(summary(model))[1], 2), 7]

    low[[ifeat]] <- summary(model, differentiation = "MD")[seq(2,
        dim(summary(model))[1], 2), 1]
    high[[ifeat]] <- summary(model, differentiation = "MD")[seq(2,
        dim(summary(model))[1], 2), 2]
} else {
    HR.factor[[ifeat]] <- summary(model)[seq(2, dim(summary(model))[1],
        2), 4]
    lower.95CI[[ifeat]] <- summary(model)[seq(2, dim(summary(model))[1],
        2), 6]
    upper.95CI[[ifeat]] <- summary(model)[seq(2, dim(summary(model))[1],
        2), 7]

    low[[ifeat]] <- summary(model)[seq(2, dim(summary(model))[1],
        2), 1]
    high[[ifeat]] <- summary(model)[seq(2, dim(summary(model))[1],
        2), 2]
}
pval[[ifeat]] <- model$stats["Score P"]

my.valid <- validate(model, B = 1000)
c[[ifeat]] <- (1 + my.valid[1, 5])/2

# Calculate corrected log-rank p-value for a continuous
```

```r
    # feature
    if (!(feat.name[ifeat] %in% c("differentiation", "histological.type",
        "T.stage"))) {
        # Load an instance of the multiple imputation datasets
        all.features <- complete(imp, 1)

        # Remove the Qatari cases
        all.features <- all.features[c(1:78), ]

        # Remove cases with met at diagnosis
        all.features <- all.features[all.features$time != 0 |
            is.na(all.features$time), ]

        all.features$histological.type <- factor(all.features$histological.type)
        all.features$T.stage <- factor(all.features$T.stage)
        all.features$differentiation <- factor(all.features$differentiation)
        all.features$met.status <- factor(all.features$met.status)

        corrected.log.rank.pval[[ifeat]] <- corrected.pval(unlist(all.features[feat.name[ifeat]]),
            all.features$time, all.features$event)
    } else {
        corrected.log.rank.pval[[ifeat]] <- pval[[ifeat]]
    }
}

# Output
output.table <- data.frame(unlist(low), unlist(high), unlist(HR.factor),
    unlist(lower.95CI), unlist(upper.95CI), unlist(pval), unlist(corrected.log.rank.pval),
    unlist(c))
colnames(output.table) <- c("Q1", "Q3", "HR.factor", "lower.95CI",
    "upper.95CI", "p.val", "log-rank.p.val", "c")
rownames(output.table) <- feat.name

# Display results
round(output.table, 3)
```

Multivariate Cox Proportional Hazards Regression Analysis

```r
# Load libraries
library(survival)
library(mice)
library(rms)

# Clear the working space
rm(list = ls())

# Define a function to merge results from different multiple
# imputation datasets
merge.result <- function(onefeat) {
    call <- match.call()
    using.Design <- FALSE
```

```
stats.ok2average <- c("linear.predictors", "fitted.values",
    "stats", "means", "icoef", "scale", "center", "y.imputed")
n.impute <- length(onefeat)
for (i in 1:n.impute) {
    f <- onefeat[[i]]

    cof <- f$coef
    v <- do.call("vcov", c(list(object = f, intercepts = "all"),
        NULL))
    if (i == 1) {
        assign <- f$assign
        ns <- num.intercepts(f)
        ik <- coef.intercepts <- NULL
        if (ns > 0) {
            ik <- attr(v, "intercepts")
            if (length(ik)) {
              if (ik == "all")
                ik <- 1:ns else if (ik == "none")
                ik <- 0
              lenik <- length(ik)
              if (length(ik) == 1 && ik == 0)
                lenik <- 0
              if (lenik != ns) {
                for (j in 1:length(assign)) assign[[j]] <- assign[[j]] -
                  (ns - lenik)
                coef.intercepts <- ik
              }
            }
        }
    }
    if (length(ik))
        cof <- c(cof[ik], cof[-(1:ns)])
    nvar0 <- length(cof)
    nvar <- nrow(v)
    if (nvar > nvar0) {
        cof <- c(cof, log(f$scale))
        names(cof) <- c(names(f$coef), if ((nvar - nvar0) ==
            1) "Log(scale)" else names(f$scale))
    }
    if (i == 1) {
        vavg <- 0 * v
        p <- length(cof)
        bar <- rep(0, p)
        vname <- names(cof)
        cov <- matrix(0, nrow = p, ncol = p, dimnames = list(vname,
            vname))
        astats <- NULL
        fitcomp <- names(f)[names(f) %in% stats.ok2average]
        if (length(fitcomp))
            for (ncomp in fitcomp) astats[[ncomp]] <- f[[ncomp]]
        if (inherits(f, "Design") | inherits(f, "rms")) {
            using.Design <- TRUE
            da <- f$Design
```

```
            }
        }
        vavg <- vavg + v
        bar <- bar + cof
        cof <- as.matrix(cof)
        cov <- cov + cof %*% t(cof)
        if (i > 1 && length(fitcomp))
            for (ncomp in fitcomp) astats[[ncomp]] <- astats[[ncomp]] +
                f[[ncomp]]
    }

    vavg <- vavg/n.impute
    bar <- bar/n.impute
    bar <- as.matrix(bar)
    cov <- (cov - n.impute * bar %*% t(bar))/(n.impute - 1)
    U <- diag(vavg)
    B <- diag(cov)
    cov <- vavg + (n.impute + 1)/n.impute * cov
    r <- diag(cov)/diag(vavg)
    names(r) <- vname
    tau <- (1 + 1/n.impute) * B/U
    missingInfo <- tau/(1 + tau)
    dfmi <- (n.impute - 1) * ((1 + 1/tau)^2)
    if (length(fitcomp))
        for (ncomp in fitcomp) f[[ncomp]] <- astats[[ncomp]]/n.impute
    if (TRUE) {
        cat("\nVariance Inflation Factors Due to Imputation:\n\n")
        print(round(r, 2))
        cat("\nRate of Missing Information:\n\n")
        print(round(missingInfo, 2))
        cat("\nd.f. for t-distribution for Tests of Single Coefficients:\n\n")
        print(round(dfmi, 2))
        if (length(fitcomp)) {
            cat("\nThe following fit components were averaged over the",
                n.impute, "model fits:\n\n")
            cat(" ", fitcomp, "\n\n")
        }
    }
    f$coefficients <- drop(bar)
    if (length(coef.intercepts))
        attr(f$coefficients, "intercepts") <- coef.intercepts
    attr(cov, "intercepts") <- ik
    f$var <- cov
    f$variance.inflation.impute <- r
    f$missingInfo <- missingInfo
    f$dfmi <- dfmi
    f$formula <- formula
    f$assign <- assign
    f$call <- call
    if (using.Design)
        options(Design.attr = NULL)
    class(f) <- c("fit.mult.impute", class(f))
    f
```

```
}

# Set a seed for a random number generator for
# reproducibility
set.seed(7887)

# Load multiple imputation data
load("multiple.imputation.RData")

# Select features. Use restricted cubic spline (rcs) to
# estimate interpolate shapes of continuous variables.

# Automatically calculated features
feat <- c("rcs(CF.smooth.muscle.ratio,3)", "rcs(CF.inflammation.ratio,3)",
    "rcs(CF.tumor.stroma.interface.ratio,3)", "rcs(CF.tumor.ratio,3)",
    "rcs(CF.stroma.ratio,3)", "rcs(CF.necrosis.ratio,3)", "rcs(AP.smooth.muscle.ratio,3)",
    "rcs(AP.inflammation.ratio,3)", "rcs(Morisita.index,3)",
    "rcs(stroma.tumor.ratio,3)", "rcs(necrosis.tumor.ratio,3)",
    "rcs(CF.smooth.muscle.ratio,3) + rcs(CF.inflammation.ratio,3)")

# Clinical features
feat2 <- c("differentiation", "histological.type", "T.stage")

for (ifeat in c(1:length(feat))) {
    onefeat <- vector("list", 100)
    for (i.impute in c(1:100)) {
        # Load an instance of the multiple imputation datasets
        all.features <- complete(imp, i.impute)

        # Remove the Qatari cases
        all.features <- all.features[c(1:78), ]

        # remove cases with met at diagnosis
        all.features <- all.features[all.features$time != 0 |
            is.na(all.features$time), ]

        all.features$met.status <- factor(all.features$met.status)
        all.features$histological.type <- factor(all.features$histological.type)
        all.features$T.stage <- factor(all.features$T.stage)
        all.features$differentiation <- factor(all.features$differentiation)

        # Survival data
        units(all.features$time) <- "Year"  # set time unit

        # Set data distribution for cph
        dd <- datadist(all.features)
        options(datadist = "dd", contrasts = c("contr.treatment",
            "contr.treatment"))


        # Fit a univariate cox proportional harzards model
        formu <- paste("Surv(time,event)~", paste(c(feat[ifeat],
            feat2), collapse = "+"), sep = "")
```

```
    onefeat[[i.impute]] <- tryCatch(cph(as.formula(formu),
        data = all.features, x = T, y = T, surv = T, time.inc = 0.1),
        error = function(e) {
            NULL
        }, warning = function(w) {
            NULL
        })  # fit logistic regression
}  # Loop through all multiple imputation datasets
onefeat <- onefeat[!sapply(onefeat, is.null)]  # remove null model
model <- merge.result(onefeat)  # merge model
model$formula <- NULL

if (feat[ifeat] == "differentiation") {
    HR.factor <- summary(model, differentiation = "MD")[seq(2,
        dim(summary(model))[1], 2), 4]
    lower.95CI <- summary(model, differentiation = "MD")[seq(2,
        dim(summary(model))[1], 2), 6]
    upper.95CI <- summary(model, differentiation = "MD")[seq(2,
        dim(summary(model))[1], 2), 7]

    low <- summary(model, differentiation = "MD")[seq(2,
        dim(summary(model))[1], 2), 1]
    high <- summary(model, differentiation = "MD")[seq(2,
        dim(summary(model))[1], 2), 2]
} else {
    HR.factor <- summary(model)[seq(2, dim(summary(model))[1],
        2), 4]
    lower.95CI <- summary(model)[seq(2, dim(summary(model))[1],
        2), 6]
    upper.95CI <- summary(model)[seq(2, dim(summary(model))[1],
        2), 7]

    low <- summary(model)[seq(2, dim(summary(model))[1],
        2), 1]
    high <- summary(model)[seq(2, dim(summary(model))[1],
        2), 2]
}

# Validate model on 1000 bootstraps
my.valid <- validate(model, B = 1000)
c <- (1 + my.valid[1, 5])/2  # concordance (AUC)

# Wald test
pval <- anova(model)[, 3]

# Output
if (feat[ifeat] %in% "rcs(CF.smooth.muscle.ratio,3) + rcs(CF.inflammation.ratio,3)") {
    output.table <- round(data.frame(low, high, HR.factor,
        lower.95CI, upper.95CI, pval[c(1, 3, 5, 6, 7)], c),
        3)
} else {
    output.table <- round(data.frame(low, high, HR.factor,
        lower.95CI, upper.95CI, pval[c(1, 3, 4, 5)], c),
```

```
                3)
    }
    # Display results
    round(output.table, 3)
}
```
